# Incremental learning of skill collections based on intrinsic motivation

**DOI:** 10.3389/fnbot.2013.00011

**Published:** 2013-07-26

**Authors:** Jan H. Metzen, Frank Kirchner

**Affiliations:** ^1^Robotics Research Group, Faculty 3 – Mathematics and Computer Science, Universität BremenBremen, Germany; ^2^Robotics Innovation Center, German Research Center for Artificial Intelligence (DFKI)Bremen, Germany

**Keywords:** hierarchical reinforcement learning, skill discovery, intrinsic motivation, life-long learning, graph-based representation

## Abstract

Life-long learning of reusable, versatile skills is a key prerequisite for embodied agents that act in a complex, dynamic environment and are faced with different tasks over their lifetime. We address the question of how an agent can learn useful skills efficiently during a developmental period, i.e., when no task is imposed on him and no external reward signal is provided. Learning of skills in a developmental period needs to be incremental and self-motivated. We propose a new incremental, task-independent skill discovery approach that is suited for continuous domains. Furthermore, the agent learns specific skills based on intrinsic motivation mechanisms that determine on which skills learning is focused at a given point in time. We evaluate the approach in a reinforcement learning setup in two continuous domains with complex dynamics. We show that an intrinsically motivated, skill learning agent outperforms an agent which learns task solutions from scratch. Furthermore, we compare different intrinsic motivation mechanisms and how efficiently they make use of the agent's developmental period.

## 1. Introduction

**Figure 1:**
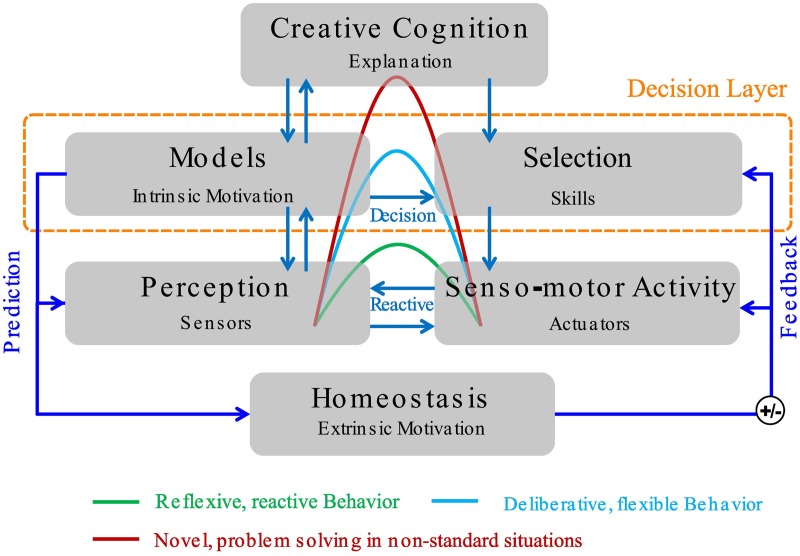
**A 3-layer control architecture, which allows the integration of reflexive reactive behaviors, more flexible decision-based behaviors, and explanation-based approaches for novel situations**. Extrinsic motivation is provided by homeostatic need regulation and prediction of fitness-enhancing events. We refer to Köhler et al. (2012) for more details.

Embodied agents like robots are used in increasingly complex, real-world domains, such as domestic and extraterrestrial settings. A simple, reactive control approach is not sufficient as it lacks the ability to predict and control the environment on larger scales of time and space. For this, agents must be able to build up competencies and knowledge about the world and store these in a convenient way so that they can be accessed fast and reliably. This requires control architectures which allow, inter alia, model-learning, predictive control, learning reusable skills, and even the integration of high-level cognitive elements. See Figure 1 for an example of such an architecture.

In this work, we focus on the middle, “decision” layer of such an architecture. One main objective on this layer is to learn a *repertoire of reusable skills*. Such skills may be the ability to reliable grasp objects, to throw, catch, or hit a ball, or to use a tool for a specific task like using a hammer to drive a nail into a wall. A repertoire of skills is useful for embodied agents which have to solve several different but related tasks during their lifetime. Instead of learning every novel task from scratch, learning skills allows that acquired capabilities are reused, i.e., transferred between tasks. Furthermore, being able to use prelearned skills may dramatically increase response times and therefore reduce the probability of system failure. One approach to skill learning is hierarchical reinforcement learning (Barto and Mahadevan, 2003), which has been applied successfully in robotic applications (see, e.g., Kirchner, 1998). Since the acquired skills shall be reusable, they should not be driven by external, task-specific reward. Instead, the agent should learn skills in a task-independent manner. In addition, an autonomous agent must decide on its own what constitutes a useful skill; this is denoted as *skill discovery*.

Existing skill discovery approaches are mostly tailored to discrete domains or to decomposing a specific task into subtasks. While the former have limited significance for continuous domains like robotics, the latter might yield skills that are task-specific and not reusable. The main contribution of this paper is a new skill discovery method which is suited for continuous domains and does not require external tasks and rewards. This method allows the agent to generate a collection of skills during a *developmental period*, in which the agent can explore freely without having to maximize external reward. The proposed skill discovery method is based on an incremental, hierarchical clustering of a learned state transition graph. This graph encodes the structure and dynamics of a domain. Densely connected subgraphs (“clusters”) of this graph correspond to qualitatively similar situations in the domain. Skills are learned for transitioning from one cluster to an adjacent one, i.e., for purposefully reaching a specific configuration of the domain.

In large domains with complex dynamics, exploring the environment, which is a prerequisite for skill discovery, is challenging by itself as is the decision whether the agent should engage in skill learning or exploration. We consider *intrinsic motivation* to reward the agent for (a) exploring novel parts of the environment and for (b) engaging in learning skills whose predictive model exhibits large error. We define novelty with regard to a set of observed states and predict skill effects based on a learned skill model which allows predicting state transitions conditioned on the specific skill.

We present an empirical analysis of the proposed approach in two continuous, high-dimensional domains with complex dynamics. We evaluate empirically to which extent the agent can benefit from reusing skills, which influence the specific skill discovery approach and the definition of intrinsic motivation have onto the agent's performance, and how the length of the agent's developmental period affects the task performance. Furthermore, we present evidence that the intrinsic motivation mechanisms can identify how much time should be spent on learning specific skills.

The paper is structured as follows: section 2 provides the necessary background and summarizes some of the most closely related works. Section 3 gives details of the main methodological contributions of this paper. In section 4, we present and discuss the results obtained in the empirical analysis. In section 5, we draw a conclusion and provide an outlook.

## 2. Background and related work

In this section, we present briefly the required background in hierarchical reinforcement learning and give a review of related works in the areas of skill discovery and intrinsic motivation.

### 2.1. Hierarchical reinforcement learning

Computational Reinforcement Learning (RL) (Sutton and Barto, 1998) refers to a class of learning methods that aims at learning behavior policies which are optimal with regard to a reward signal, through interaction with an environment. The most popular problem class for RL are Markov Decision Processes (MDPs). An MDP *M* can be formalized as a 4-tuple *M* = (*S, A, P*^*a*^_*ss*′_, *R*^*a*^_*ss*′_) where *S* is a set of states of the environment, *A* is a set of actions, *P*^*a*^_*ss*′_ = *P*(*s*_*t* + 1_ = *s*′| *s*_*t*_ = *s, a*_*t*_ = a) is the 1-step state transition probability also referred to as the “dynamics,” and *R*^*a*^_*ss*′_ = *E*{*r*_*t* + 1_| *s*_*t*_ = *s, a*_*t*_ = *a, s*_*t* + 1_ = *s*′} is the expected reward. In RL, these quantities are usually unknown to the agent but can be estimated based on samples collected during exploration. If both *S* and *A* are finite, we call *M* a discrete MDP, otherwise we call it a continuous MDP. The goal of RL is to learn without explicit knowledge of *M* a policy π^*^ such that some measure of the long-term reward is maximized. Popular approaches to RL include value-function based methods, which are based on approximating the optimal action-value function Q^*^(*s, a*) = ∑_*s*′_ *P*^*a*^_*ss*′_[*R*^*a*^_*ss*′_ + γmax_*a*′_Q^*^(*s*′, *a*′)], where γ ∈ [0, 1] is a discount factor, and direct policy search methods, which search directly in the space of policies based on, e.g., evolutionary computation (Whiteson, 2012).

This paper focuses on learning *skills* using Hierarchical RL (Barto and Mahadevan, 2003). In Hierarchical RL, behavior is not represented by a monolithic policy but by a hierarchy of policies, where policies on the lowest layer correspond to simple skills and policies on higher layer are based on these skills and represent more complex behavior. One popular approach to Hierarchical RL is the *options framework* (Sutton et al., 1999). An option *o* is the formalization of a temporally extended action or skill and consists of three components: the option's initiation set *I*_*o*_ ⊂ *S* that defines the states in which the option may be invoked, the option's termination condition β_*o*_: *S* → [0, 1] which specifies the probability of option execution terminating in a given state, and the option's policy π_*o*_ which defines the probability of executing an action in a state under option *o*. In the options framework, a policy on a higher layer may in any state *s* decide not solely to execute a primitive action but also to call any of the lower-layer options for which *s* ∈ *I*_*o*_. If an option is invoked, the option's policy π_*o*_ is followed for several time steps until the option terminates according to β_*o*_. The option's policy π_*o*_ is defined relative to an option-specific “pseudo-reward” function *R*_*o*_ that rewards the option for achieving the skill's objective. Skill learning denotes learning π_*o*_ given *I*_*o*_, β_*o*_, and *R*_*o*_. *Skill discovery*, on the other hand, requires choosing appropriate *I*_*o*_, β_*o*_, and *R*_*o*_ for a new option *o*. Skill discovery is very desirable since the quantities *I*_*o*_, β_*o*_, and *R*_*o*_ need not be predefined but can be identified by the agent itself and thus, skill discovery increase the agent's autonomy. We give a review of related works in the next section.

### 2.2. Skill discovery

Most prior work on autonomous skill discovery is based on the concept of *bottleneck areas* in the state space. Informally, bottleneck areas have been described as the border states of densely connected areas in the state space (Menache et al., 2002) or as states that allow transitions to a different part of the environment (Şimşek and Barto, 2004). A more formal definition is given by Şimşek and Barto (2009), in which bottleneck areas are states that are local maxima of betweenness—a measure of centrality on graphs—on the transition graph. Once bottleneck areas have been identified, typically one (or several) skills are defined that try to reach this bottleneck, i.e., that terminate with positive pseudo-reward if the bottleneck area is reached, can be invoked in a local neighborhood of the bottleneck, and terminate with a negative pseudo-reward when departing too far from the bottleneck.

Since betweenness requires complete knowledge of the transition graph and is computationally expensive, several heuristics have been proposed to identify bottlenecks. One class of heuristics are *frequency-based approaches* that compute local statistics of states like diverse density (McGovern and Barto, 2001) and relative novelty (Şimşek and Barto, 2004). An other class of heuristics that is typically more sample-efficient are *graph-based approaches* which are based on estimates of the domain's state transition graph. Graph-based approaches to skill discovery aim at partitioning this graph into subgraphs which are densely connected internally but only weakly connected with each other. Menache et al. (2002) propose a top–down approach for partitioning the global transition graph based on the max-flow/min-cut heuristic. Şimşek et al. (2005) follow a similar approach but partition local estimates of the global transition graph using a spectral clustering algorithm and use repeated sampling for identifying globally consistent bottlenecks. Mannor et al. (2004) propose a bottom–up approach that partitions the global transition graph using agglomerative hierarchical clustering. Metzen (2012) proposes an extension of this approach called OGAHC. OGAHC is incremental and can thus be performed several times during the learning process. A further approach for identifying bottlenecks is to monitor the propagation of Q-values in the planning phase of a model-based RL architecture. For instance, Kirchner and Richter (2000) have shown that the so-called significance values become large close to bottlenecks of the domain.

Relatively few works on autonomous skill discovery in domains with continuous state spaces exist. Frequency-based approaches do not easily generalize to such domains since their statistics are typically related to individual states and there exist infinitely many such states in continuous domains. Similarly, the 1-to-1 relationship between states and graph nodes hinders the direct applicability of graph-based approaches. Mannor et al. (2004) have evaluated their agglomerative hierarchical clustering approach in the mountain car domain by uniformly discretizing the state space. However, this uniform discretization is suboptimal since it suffers from alignment effects and the “curse of dimensionality.” Learning an adaptive discretization in the form of a transition graph that captures the domain's dynamics using the FIGE heuristic (see section 3.2) is shown to perform considerably better (Metzen, in press). However, FIGE is a batch method and requires that skill discovery is performed at a prespecified point in time.

One skill discovery method that has been designed for continuous domains is “skill chaining” (Konidaris and Barto, 2009). Skill chaining produces chains (or more general: trees) of skills such that each skill allows reaching a specific region of the state space, such as a terminal region or a region where an other skill can be invoked. In which region of the state space a skill can be invoked depends mainly on the representability and learnability of the skill in the specific learning system and not directly on concepts like bottlenecks or densely connected regions. Skill chaining requires to specify an area of interest (typically the terminal region of the state space) which is used as target for the skill at the root of the tree. For multi-task domains with several goal regions or domains without a goal region, it is unclear how the root of the skill tree should be chosen.

### 2.3. Lifelong learning and intrinsic motivation

Thrun (1996) suggested the notion of *lifelong learning* in the context of supervised learning for object recognition. In lifelong learning, a learner experiences a sequence of different but related tasks. Due to this relatedness, learned knowledge can be transferred across multiple learning tasks, which can allow generalizing more accurately from less training data. The concept of lifelong learning was extended to RL by, e.g., Sutton et al. (2007). In RL, lifelong learning is often combined with *shaping*, which denotes a process where a trainer rewards an agent for a behavior that progresses toward a desired target behavior which solves a complex task. Thus, shaping can be seen as a training procedure for guiding the agent's learning process. Shaping was originally proposed in psychology as an experimental procedure for training animals (Skinner, 1938) and has been adopted for training of artificial systems later on (Randløv Alstrøm, 1998). One disadvantage of shaping is that an external teacher is required which selects tasks of a specific complexity carefully by taking the current developmental state of the agent into account. This reduces the agent's autonomy.

A different approach to lifelong learning, in which no external teacher is required, is to provide the agent with a means for *intrinsic motivations*. The term “intrinsically motivated” stems from biology and one of its first appearances was in a paper by Harlow (1950) on the manipulation behavior of rhesus monkeys. According to Baldassarre (2011) “extrinsic motivations guide learning of behaviors that directly increase (evolutionary) fitness” while “intrinsic motivations drive the acquisition of knowledge and skills that contribute to produce behaviors that increase fitness only in a later stage.” Thus, similar to shaping, intrinsic motivations contribute to learning not as a learning mechanism *per se*, but rather as a guiding mechanism which guides learning mechanisms to acquire behaviors that increase fitness. According to Baldassarre “(intrinsic motivations) drive organisms to continue to engage in a certain activity if their competence in achieving some interesting outcomes is improving, or if their capacity to predict, abstract, or recognize percepts is not yet good or is improving….” Accordingly, learning signals produced by intrinsic motivations tend to decrease or disappear once the corresponding skill is acquired.

Computational approaches to intrinsic motivation [see Oudeyer and Kaplan (2007) for a typology] have become popular in hierarchical RL in the last decade resulting in the area of Intrinsically Motivated Reinforcement Learning (IMRL) (Barto et al., 2004). Work on intrinsic motivation in RL, however, dates back to the early 1990s (Schmidhuber, 1991). IMRL often employs a *developmental setting* [see, e.g., Stout and Barto (2010) and Schembri et al. (2007)], which differs slightly from the usual RL setting where the objective is to maximize the accumulated external reward. In the developmental setting, the agent is given a developmental period, which can be considered as its “childhood,” in which no external reward is given to the agent. This allows the agent to explore its environment freely without having to maximize the accumulated reward (exploitation). On the other hand, the agent is not guided by external reward but needs to have a means for intrinsic motivation. The objective in the developmental setting is to learn skills which allow to quickly learn high-quality policies in tasks that are later on imposed onto the agent. Thus, the objective can be seen as a kind of optimal exploration for skill learning, in contrast to finding the optimal balance between exploration and exploitation as in usual RL. Different mechanisms for intrinsic motivation have been proposed. A complete review is beyond the scope of this paper, we discuss a selected subset of methods and refer to Oudeyer et al. (2007) for a review.

Barto et al. (2004) investigate how a hierarchically organized collection of reusable skills can be acquired based on intrinsic reward. Their notion of intrinsic reward is based on the novelty response of dopamine neurons. More precisely, the intrinsic reward for a *salient event* is proportional to the error of predicting this salient event based on a learned skill model for this event. This skill model is not only a passive model of the environment but it is also dependent on the agent's action preferences. As a result of the intrinsic reward, once the agent encounters an unpredicted salient event, it is driven to attempt to achieve this event until it has learned to predict it satisfyingly.

Oudeyer et al. (2007) propose an intrinsic motivation system that encourages the robot to explore situations in which its current *learning progress* is maximized. More specifically, the robot obtains a positive intrinsic reward for situations in which the error rate of internal predictive models decreases and a negative one for situations in which it increases. Thereby, the robot focuses on exploring situations whose complexity matches its current stage of development, i.e., situations which are neither too complex (too unpredictable) nor too simple (too predictable).

Hester and Stone (2012) propose a model-based approach for a developing, curious agent called TEXPLORE-VANIR. This approach uses two kinds of intrinsic reward that are derived from the learned model. The first one rewards the agent for exploring parts of the environment for which the variance in the model's prediction is large while the second one rewards the agent for exploring parts of the environment that are *novel* to the agent. The authors show empirically that these intrinsic rewards are helpful for an agent in a developmental setting. Furthermore, the intrinsic rewards also improve the performance of an agent faced with an external task from the very beginning by providing a reasonable explorative bias.

Stout and Barto (2010) propose “competence progress motivation,” which generates intrinsic rewards based on the skill competence progress, i.e., how strongly the agent's competence to achieve self-determined goals progresses. The authors show on a simple problem that the approach is able to focus learning efforts onto skills that are neither too simple not too difficult at the moment. While the authors predefine the set of skills that shall be learned, they note that “identifying what skills should be learned is a very important problem and one that a complete motivational system would address.” This problem is addressed in this paper.

Note that intrinsic motivations need not be the only source of motivation in a biologically-inspired robotic control architecture such as the one shown in Figure 1; rather, extrinsic motivations based on homeostatic need regulation and prediction of fitness-enhancing visceral-body changes (compare Baldassarre, 2011) should be taken into account as well. However, since we focus on the “decision” layer of the architecture, we do not consider these kinds of motivations in detail here.

## 3. Methods

In this section, we present an architecture for an IMRL-agent and propose new methods for skill discovery and intrinsic motivation.

### 3.1. Agent architecture

We consider an agent situated in an environment with state space *S* and action space *A*. We are particularly interested in problems where the state and/or the action space are continuous, more specifically where *S* ⊆ ℝ^*n*_*s*_^ and/or *A* ⊆ ℝ^*n*_*a*_^. We assume that the state transitions (the effects of executing an action in a state) have the Markov property. During its lifetime, the agent may be faced with different tasks in this environment; we assume that each task 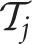 is specified by a reward function ℛ_*j*_ = *E*(*r*_*t* + 1_| *s*_*t*_ = *s, a*_*t*_ = *a, s*_*t* + 1_ = *s*′) and the agent needs to maximize a long-term notion of this reward. Note that each task thus corresponds to a MDP 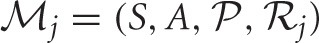, where all tasks share *S, A*, and 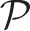.

We adopt the developmental setting of IMRL (see section 2.3, i.e., we assume that the agent has a developmental period before it is faced with an external task. The agent-environment interaction during the developmental period can be modeled as an MDP without reward 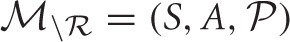. Thus, we implicitly assume that the developmental period takes place in the same environment where the agent has to solve tasks later on, i.e., we assume *S, A*, and 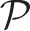 to be identical. While no external objective is imposed on the agent, the agent should use the developmental period nevertheless for learning a repertoire of skills *O* that can later on help in solving tasks 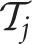. Furthermore, we do not provide the agent with a set of subgoals or salient events but require the agent to identify these on its own.

**Figure 2:**
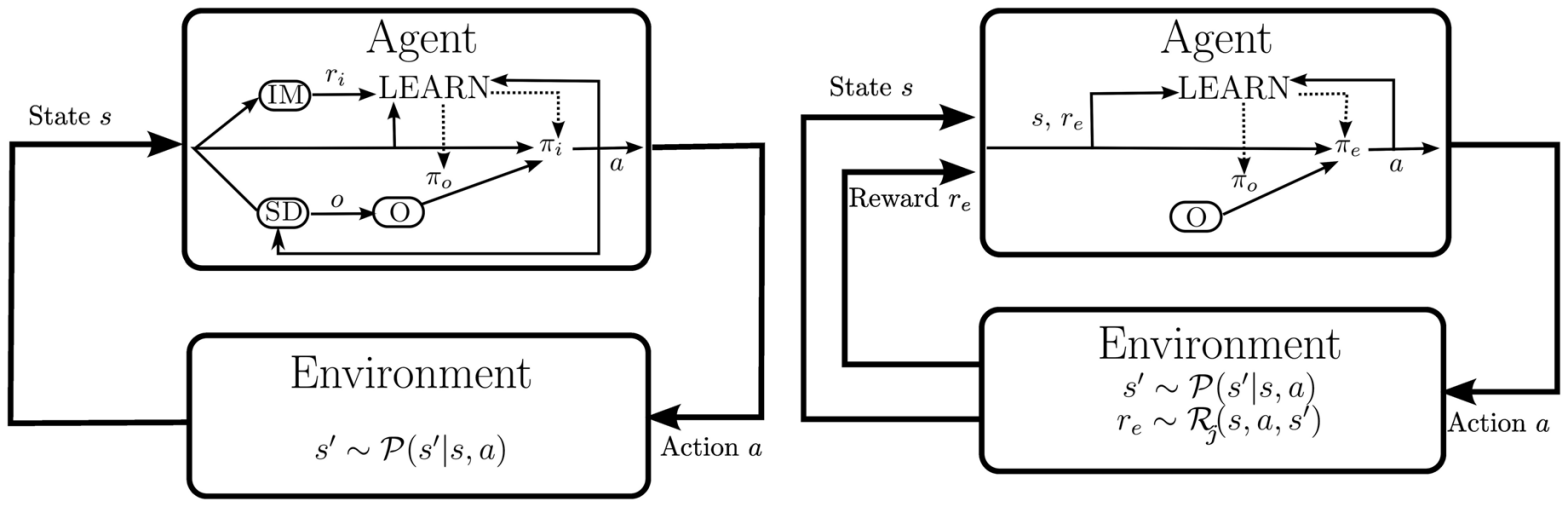
**Left plot:** Agent architecture employed during the developmental period. No external reward is provided but the motivational system IM creates an intrinsic reward *r*_*i*_. In parallel, new skills *o* are identified using the skill discovery module SD and added to the skill pool *O*. The policy π_*i*_ selects skills according to their intrinsic reward; both π_*i*_ and the policy π_*o*_ of the active skill are learned. **Right plot:** Agent architecture for learning to solve external tasks 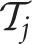. A hierarchical policy π_*e*_ is learned based on the external reward *r*_*e*_ using the fixed set of skills *O*. The policy π_*o*_ of the active skill is also improved.

For this, two questions need to be addressed: (a) how are useful and task-independent skills identified autonomously? and (b) how does the agent select actions and skills when no external reward is available? We address these questions in section 3.2 and section 3.3, respectively. For now, we assume that two modules for intrinsic motivation (IM) and skill discovery (SD) exist where IM generates an intrinsic reward signal *r*_*i*_ which the agent uses in place of external reward and SD identifies new skills which are added to the skill repertoire *O* and whose policy is learned later on by the agent using option learning. The agent's internal architecture during its developmental period is depicted in the left diagram in Figure 2.

Once an external task 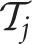 is imposed onto the agent, the intrinsic reward and the skill discovery modules are disabled, and the agent learns a hierarchical policy π_*e*_ over the set of discovered skills *O* that maximizes the external reward *r*_*e*_ (see right diagram in Figure 2). Note that the agent continues to learn option policies π_*o*_ based on experience collected; however, the external reward is ignored in skill learning such that options remain task-independent.

### 3.2. Iterative graph-based skill discovery

A skill discovery method which can be used in the outlined architecture needs to exhibit the following properties: (1) it needs to be suited for continuous domains, (2) it needs to be incremental, i.e., the agent must be able to identify new skills at any time and not just once after some predefined amount of experience was collected, and (3) it must not require that an external reward signal or a goal region of a task exist. None of the methods discussed in section 2.2 fulfills all these requirements. In this work, we propose IFIGE, an incremental extension of FIGE (Metzen, in press), which is combined with an extension of OGAHC (Metzen, 2012) to continuous domains. This combination fulfills all of requirements given above. The key idea of the approach is that a transition graph, which captures the domain's dynamics, is learned incrementally from experience using FIGE and that the learned graphs are clustered into densely connected subgraphs using OGAHC. These clusters correspond to subareas of the domain's state space and the connections between these subparts form bottlenecks of the domain. Learning skills which allow traversing these bottlenecks is a common approach to skill discovery in discrete domains (compare section 2.2).

#### 3.2.1. Incremental transition graph estimation in continuous domains

A transition graph *G* = (*V, E, w*) can be seen as a model of the domain's 1-step state transition probability (the domain's “dynamics”), where the nodes *v* ∈ *V* represent “typical” states of the domain and edges (*v, v*′)_*a*_ ∈ *E* represent possible transitions in the domain under a specific action *a*. The edge weights *w* encode the corresponding probabilities *P*^*a*^_*vv*′_. In a model-free setting, *G* needs to be learned from experience. While this is straightforward in domains with discrete state space, it is more challenging in continuous domains. Force-based Iterative Graph Estimation (FIGE) is an heuristic approach to this problem with a solid theoretical motivation. FIGE learns transition graphs of size *v*_num_ from a set of state transitions *T* = {(*s*_*i*_, *a*_*i*_, *s*′_*i*_)}^*n*^_*i* = 1_ that have been experienced by the agent while acting in the domain. The transition graph is considered to be a generative model of state transitions and FIGE aims at finding graph node positions *V* which maximizes the likelihood of the observed transition (Metzen, in press).

FIGE is summarized in Algorithm 1: the set of graph nodes *V* with cardinality |*V*| = *v*_num_ is initialized such that it covers the set of states contained in *T* uniformly by, e.g., maximizing the distance of the closest pair of graph nodes (line 2). Afterwards, for *K* iterations, the graph nodes are moved according to two kind of “forces” that act on them: the “sample representation” force (lines 5, 6) pulls each graph node *v* to the mean of all states *S*^*V*^ for which it is responsible, i.e., the states *s* for which it is the nearest neighbor NN_*V*_(*s*) in *V*. Thus, this force corresponds to an intrinsic k-means clustering of the observed states. The “graph consistency” force (lines 7–9) pulls each graph node *v* to a position where for all (*s, a, s*′) ∈ *T* with NN_*V*_(*s*) = *v* there is a vertex *v*′ such that *v*′−v is similar to *s*′−s, i.e., both vectors are close to parallel. Thus, this force encourages node positions which can represent the domain's dynamics well. The nodes are then moved according to the two forces (line 11), where the parameter α_*i*_ ∈ (0, 1] controls how greedily the node is moved to the position where the forces would become minimal. In order to ensure convergence of the graph nodes, α_*i*_ should go to 0 for *i* approaching *K*. An edge labeled with action *a* is added between two nodes *v* and *v*′ if there exists at least one transition (*s, a, s*′) ∈ *T* with *v* being the nearest neighbor of *s* in *V* and *v*′ being the nearest neighbor of *s*′ in *V* (line 14). Furthermore, the edge weights are chosen as the empirical transition probabilities ${\hat{P}}_{vv^{\prime }}^{a}$ from node *v* to *v*′ under action *a* (line 15). For details and a derivation of FIGE, we refer to Metzen (in press).

**Algorithm 1:**
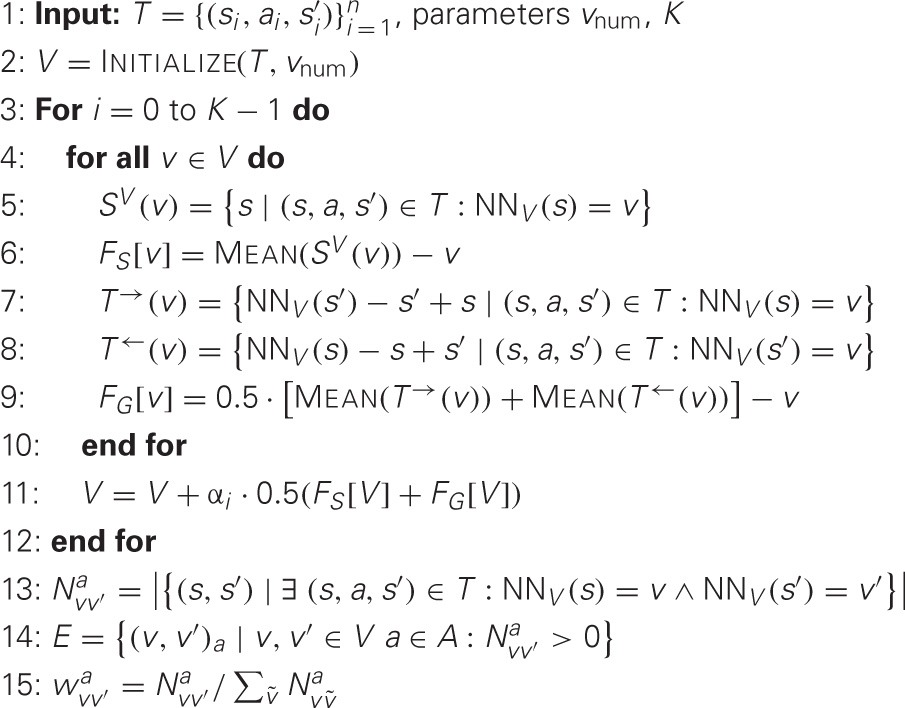
Force-based Iterative Graph Estimation (FIGE)

The main drawbacks of FIGE are that the number of nodes of the transition graph need to be pre-specified and that FIGE is a batch algorithm and thus not well suited for incremental skill discovery. We present now Incremental FIGE (IFIGE) which does not suffer from these problems. IFIGE updates the graph's node positions after every experienced transition. Furthermore, IFIGE stores for every graph node *v* a set of exemplar states *S*_*v*_ = {*s*_*i*_ | *i* = 1,…, *n*_*v*_} and exemplar transitions *T*_*v*_ = {(*s*_*i*_, *a*_*i*_, *s*′_*i*_) | *i* = 1,…, *n*_*v*_}, with all *s*_*i*_ being “similar” to *v* and *n*_*v*_ being set typically to 25.

IFIGE starts with a single graph node *V* = {*s*_0_} and *S*_*s*_0__ = *T*_*s*_0__ = ∅, where *s*_0_ is the start state. For any encountered transition (*s, a, s*′), the most similar graph node *v* = NN_*V*_(*s*), i.e., the nearest neighbor of *s* in *V*, is determined, *s* is added to the set of state exemplars *S*_*v*_, and (*s, a, s*′) to *T*_*v*_. If the size of *S*_*v*_ or *T*_*v*_ exceeds *n*_*v*_, old exemplars are deleted. Afterwards, the position of vertex *v* is updated using lines 5–9 of Algorithm 1 for *T* = *T*_*v*_. This changes the position of *v*; thus, IFIGE checks afterwards for all state exemplars in *S*_*v*_ and transition exemplars in *T*_*v*_ whether any other node in *V* would be a better representative and moves the exemplars if required. Afterwards, IFIGE checks whether *v* is responsible for a too large area of the state space by computing the distance of the farthest pair in *S*_*v*_. If this distance is above a threshold ζ, *v* is removed from *V* and two new nodes *v*_1_ and *v*_2_ are added to *V*. *v*_1_ and *v*_2_ are chosen as the cluster centers of a *k*-means clustering of *S*_*v*_ for *k* = 2. *S*_*v*_ and *T*_*v*_ are split into two subsets accordingly. Splitting nodes ensures that the number of graph nodes grows with the size of the state space explored by the agent.

When the current transition graph needs to be generated for skill discovery, IFIGE adds for all graph nodes *v* and any transition (*s, a, s*′) ∈ *T*_*v*_ an edge between *v* and *v*′ = NN_*V*\{*v*}_(*s*′) for action *a*. Edge weights are determined by counting the frequencies of edges from *v* to *v*′ relative to all edges starting from *v*.

#### 3.2.2. Online graph-based agglomerative hierarchical clustering

Based on the transition graph, we identify task-independent and thus reusable skills using “Online Graph-based Agglomerative Hierarchical Clustering” (OGAHC). We give a brief summary of OGAHC and discuss how it can be extended to continuous domains; for more details we refer to the original publication (Metzen, 2012). OGAHC identifies skills by computing a partition P^*^ of the nodes *V* of a given transition graph *G* with respect to a prespecified linkage criterion *l*. Formally: 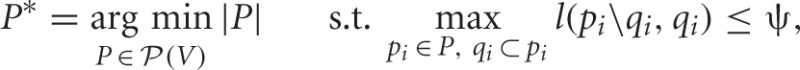


with 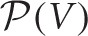 being the set of all possible partitions of *V* and ψ being a threshold which controls the granularity of the partition, i.e., the number of elements of the partition (called “cluster”). The aim is thus to compute a partition of the graph nodes with minimal cardinality such that the linkage between any pair of clusters of the partition is small, i.e., below ψ. Since this problem is 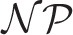-hard, we use agglomerative hierarchical clustering as proposed by Mannor et al. (2004) for identifying an approximately optimal solution. As proposed by Şimşek et al. (2005), we use the normalized cut ${\hat{N}}_{\text{cut}}$ as linkage. The ${\hat{N}}_{\text{cut}}$ of two disjoint subgraphs *A, B* ⊂ *G* is an approximation of the probability that a random walk on *G* transitions in one time step from a state in subgraph *A* to a state in subgraph *B* or vice versa. Thus, we identify areas of the state space (corresponding to clusters of the graph) such that a randomly behaving agent would very unlikely leave one of these areas.

The connections of these clusters form *bottlenecks* of the graph and thus also of the domain. OGAHC creates one skill prototype for each pair of clusters *c*_1_, *c*_2_ ∈ P^*^ which are connected in *G*; this skill can be invoked any state *s* with NN_*V*_(*s*) ∈ *c*_1_ and terminates in any state with NN_*V*_(*s*) ∉ *c*_1_. It terminates successfully if NN_*V*_(*s*) ∈ *c*_2_ and fails otherwise. Thus, the skill's objective is to guide the agent through one of the domain's bottlenecks from the area corresponding to cluster *c*_1_ to the area of cluster *c*_2_.

Since the transition graph, which is the basis for OGAHC, is learned from experience and thus changes over time, performing the clustering only once is problematic: performing it early might result in a bad clustering of the domain since the transition graph might be inaccurate, while performing it late can overly increase the amount of experience the agent requires for skill discovery. Thus, it is desirable to perform the clustering several times during learning. For this, OGAHC assumes “dense local connectivity in the face of uncertainty,” which prevents premature identification of bottlenecks and the corresponding skills, and adds constraints to the clustering process, which ensure that subsequent partitions remain consistent with prior ones. These constraints enforce that graph nodes that have been assigned to different clusters in one invocation of OGAHC remain in different clusters in later invocations.

The main hindrance of OGAHC in domains with continuous state space is that the constraints are based on the assumption that the graph nodes do not change over time. This is not the case when OGAHC is applied on top of IFIGE. This problem can be alleviated by adapting the current partition to the changes in the graph prior to any invocation of OGAHC. For this, let P^*^(*V*) be the partition of the graph nodes *V* of the last invocation of OGAHC and *V*′ the current node positions. We extend P^*^(*V*) to a (pre-)partition *P*_pre_ of *V*′ by assigning nodes *v*_*a*_′, *v*_*b*_′ ∈ *V*′ to the same cluster if NN_*V*_(*v*_*a*_′) and NN_*V*_(*v*_*b*_′) are in the same cluster in P^*^(*V*). Now, OGAHC can be invoked with the usual constraints that nodes which are in different clusters in *P*_pre_(*V*′) must be in different clusters in P^*^(*V*′). For nodes *v*′ ∈ *V*′ whose nearest neighbor NN_*V*_(*v*′) is very different from *v*′, this constraint is relaxed, i.e., these nodes can be assigned to any cluster in P^*^(*V*′). This corresponds to a situation where the agent has visited a particular area of the state space for the first time and the prior invocations of OGAHC put no restrictions on the bottlenecks in this novel part.

### 3.3. Intrinsic motivation

In the context of this paper, intrinsic motivation refers to the process of mapping a transition from state *s* under option *o* to successor state *s*′ onto an intrinsic reward *r*_*i*_. We investigate two different intrinsic motivation mechanisms, one based on the *novelty* of a state under a skill and one based on the *prediction error* of a learned skill model.

For the novelty based motivation criterion, the agent stores for each option *o* the states it has encountered under this option so far in the set *S*_*o*_^1^. When transitioning to state *s*′ under option *o*, the intrinsic reward is computed via

$$r_{i}=-\sum_{j\in {\text{ NN}}_{S_{o}}^{10}(s^{\prime })}\exp\left(-\frac{||s^{\prime }-s_{j}|{|}_{2}^{2}}{b^{2}}\right),$$

where NN^10^_*S*_*o*__(*s*′) denotes the indices of the 10-nearest neighbors of *s*′ in *S*_*o*_ and *b* is a domain-dependent scale parameter. Thus, the intrinsic reward is upper-bounded by 0 with values close to 0 if the 10 nearest neighbor are very different (large euclidean distance) from *s*′ and very small values when *s*′ is similar to several states in *S*_*o*_. Thus, the novelty criterion discourages to execute options in regions of the state space where this option has been executed already several times. This mechanism is similar to the mechanism proposed by Hester and Stone (2012); however, in contrast to their work, it is also suited for domains with continuous state spaces.

For the prediction error criterion, the agent learns for each option a model ${\hat{P}}_{o}$ that predicts the successor state of states *s* when following option *o*. The intrinsic reward is determined based on the error of the model's prediction via

$$r_{i}=-1+\tanh(\sigma ||s^{\prime }-{\hat{P}}_{o}(s)|{|}_{2}^{2}),$$

where σ is a domain-dependent scale parameter. The intrinsic reward *r*_*i*_ is large (close to 0) when the difference of predicted successor ${\hat{P}}_{o}(s)$ and actual successor *s*′ is large. The intrinsic reward becomes small (close to −1) when the model correctly predicts the effect of executing option *o* in state *s*. Thus, the prediction error criterion encourages to execute options whose effects are unknown or unpredictable in the current area of the state space. Note that in contrast to the novelty criterion, for the prediction error criterion the intrinsic reward in a state depends on the option's policy.

The option model ${\hat{P}}_{o}$ stores internally a set *T*_*o*_ = {(*s*_*j*_, *s*′_*j*_)} of transitions encountered under option *o*. The model's prediction is based on 10-nearest neighbors regression:

$$P_{o}(s)=s+\frac{1}{10}\sum_{j\in {\text{ NN}}_{T_{o}}^{10}(s)}\left({s^{\prime }}_{j}-s_{j}\right),$$

where NN^10^_*T*_*o*__(*s*) denotes the indices of the 10-nearest neighbors of *s* in the start states in *T*_*o*_. If the size of *T*_*o*_ exceeds a threshold (in the experiments 2500) and a transition from *s* to *s*′ is added, the oldest transition among NN^10^_*T*_*o*__(*s*) is removed. This is required to keep the memory consumption limited and, more importantly, to track the non-stationarity in the target function that is induced by learning the option *o* concurrently and thus changing *o*′s policy.

## 4. Results

In this section, we present an empirical evaluation of the proposed methods in two continuous and challenging RL benchmark domains. We evaluate both the behavior of the agent during the developmental period and its performance in external tasks. We have chosen these benchmark domains since they allow other researchers to compare their methods easily to our results.

### 4.1. 2D multi-valley

#### 4.1.1. Problem domain

**Figure 3:**
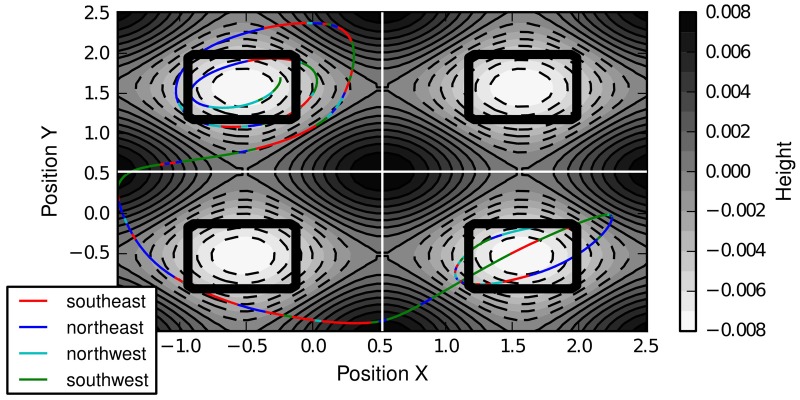
**2D Multi-Valley domain**. Gray-scale contours depict the height of the two-dimensional surface. The black boxes denote the target regions of the different tasks and the white lines the boundaries of the valleys. Shown is one example trajectory with color-coded actions.

The 2D Multi-Valley environment (see Figure 3) is an extension of the basic mountain car domain. The car the agent controls is not restrained to a one-dimensional surface, however, but to a two-dimensional surface. This two-dimensional surface consists of 2 × 2 = 4 valleys, whose borders are at (π/6 ± π/3, π/6 ± π/3). The agent observes four continuous state variables: the positions in the two dimensions (*x* and *y*) and the two corresponding velocities (*v*_*x*_ and *v*_*y*_). The agent can choose among the four discrete actions northwest, northeast, southwest, southeast which add (±0.001, ±0.001) to (*v*_*x*_, *v*_*y*_). In each time step, due to gravity 0.004cos(3x) is added to *v*_*x*_ and 0.004cos(3y) to *v*_*y*_. The maximal absolute velocity in each dimension is restrained to 0.07. The four valleys correspond naturally to clusters of the domain since transitioning from one valley to the other is unlikely under random behavior, i.e., represents a bottleneck. Thus, we would expect that one skill is created for each combination of adjacent valleys.

#### 4.1.2. Developmental period

During its developmental period, the agent can explore the domain freely while engaging in skill discovery and following its intrinsic motivations. Initially, the agent has only a single option *o*_*e*_ in its skill pool *O*, which can be invoked in any state of the environment, i.e., *I*_*o*_*e*__ = *S*, and terminates with probability β_*o*_*e*__(*s*) = 0.05. This option can be considered to be the agent's exploration option, which can always be invoked if the agent prefers to explore the environment over learning a specific skill. We set the greediness of IFIGE to α_*i*_ = 0.25 and the split node distance to ζ = 0.3. For OGAHC, we set the maximal linkage to ψ = −0.075 and performed skill discovery every 5000 steps.

Each option's value function has been represented by an CMAC function approximator consisting of 10 independent tilings with 7^2^ · 5^2^ tiles, where the higher resolutions have been used for the *x* and *y* dimensions. The pseudo-reward for each option's policy has been set to *r*_*o*_ = −1 for each step and *r*_*o*_ = −1000 if an option terminates unsuccessfully, i.e., leaves its initiation set *I*_*o*_ without reaching its goal cluster *c*_2_. Value functions have been initialized to −100. For learning the higher-level policy π_*i*_, a lower resolution of 5^2^ · 3^2^ tiles has been used and the value functions have been initialized to 0. The discounting factor has been set to γ = 0.99 and all policies were ϵ-greedy with ϵ = 0.01. The value functions were learned using Q-Learning and updated only for currently active options with a learning rate of 1. The scale-parameters of the intrinsic motivation mechanisms have been set to *b* = 0.1 (novelty) and σ = 10^4^ (prediction error). All parameters have been chosen based on preliminary investigations.

**Figure 4:**
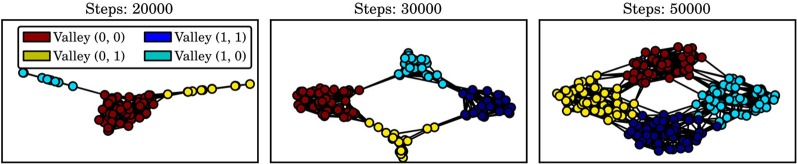
**Two-dimensional embedding (determined using Isomap) of the learned transition graphs**. Densely connected subgraphs correspond to the four valleys.

Figure 4 shows the transition graphs generated by IFIGE after 20,000, 30,000, and 50,000 developmental steps. The two-dimensional embeddings of the graphs have been determined using Isomap (Tenenbaum et al., 2000). The four valleys of the domain clearly correspond to four densely connected subgraphs of the transition graph. The figure also shows that it would be difficult to determine a single point in time at which skill discovery should be performed: for instance, are the valleys (0, 1) and (1, 0) explored sufficiently after 30,000 steps to perform graph clustering? Since skill discovery with OGAHC is incremental, i.e., can be performed several times during learning, this choice need not be made.

**Figure 5:**
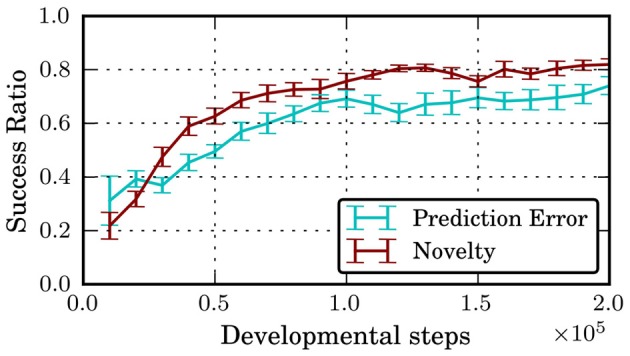
**Success ratio of learned skills over developmental period**. Shown are mean and standard error of mean averaged over 10 independent runs.

Figure 5 shows the success ratio, i.e., how often a skill reaches its goal cluster, of the skills discovered during the developmental period. Initially, skills are unlikely to reach their goal area, with success ratios of approximately 0.25. Under both intrinsic motivation systems, the agent invests time in learning skill policies and the success ratio increases to 0.7 for the prediction error and 0.8 for the novelty criterion after approximately 10^5^ steps of development. Note that success ratios of 1.0 are not possible since for some states in *s* ∈ *I*_*o*_, there is no way of reaching the option's goal area without leaving the initiation set, e.g., when the agent is moving with high velocity in the direction of the wrong neighbor valley. A possible explanation for the different performance under the two motivational systems is given below.

**Figure 6:**
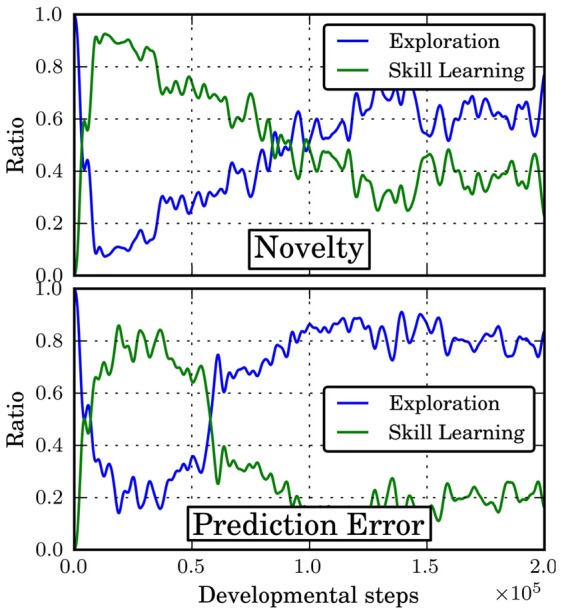
**Ratio of skill learning to exploration during developmental period**. Shown is mean over 10 independent runs.

Figure 6 shows the ratio of selecting the option *o*_*e*_ (“Exploration”) or any of the other, discovered options in *O* (“Skill Learning”) under the policy π_*i*_ for different intrinsic motivations. Initially, no skills have been discovered and the agent thus has to explore. Once the first skills have been discovered, the agent focuses onto learning these skills. Over time, as the skill policies converge, a better predictive model for these skills can be learned. Similarly, the more time is spend on learning a skill, the less novel states are encountered under this skill. Accordingly, both intrinsic motivation mechanisms reduce the ratio of skill learning and focus on exploration again in order to discover new skills. Note that at this point in time, there are no further skills to be discovered in this domain but this is unknown to the agent.

In general, the prediction error-based motivation chooses the exploration option more often and reduces skill learning more abruptly than the novelty criterion. This can be explained by the fact that the exploration policy changes more strongly over time and it is thus harder to learn a model of this option. Once the policies of the other skills have settled, they are chosen only rarely. However, the results in Figure 5 suggest that this happens too early as the final “fine-tuning” of the skill policies is not finished and the success ratio is smaller than for the novelty criterion. Thus, the results indicate that using the prediction error for intrinsic motivation can be detrimental in situations where different option policies explore to different degrees since the prediction error criterion will favor the options with stronger exploration. Thus, it is recommended to base motivation on criteria like novelty or on the *change* of prediction error rather than on the error itself.

#### 4.1.3. Task performance

In its “adulthood,” the agent is faced with a multi-task scenario: in each episode, the agent has to solve one out of 12 tasks. Each task is associated with a combination of two distinct valleys; e.g., in task (0, 1) the agent starts in the floor^2^ of valley 0 and has to navigate to the floor of valley 1 and reduce its velocity such that ‖(*v*_*x*_, *v*_*y*_)‖_2_ ≤ 0.03. In each time step, the agent receives an external reward of *r*_*e*_ = −1. Once a task is solved, the next episode starts with the car remaining at its current position and one of the tasks that starts in this valley is drawn at random. Episodes have been interrupted after 10^4^ steps without solving the task and a new task was chosen at random. The current task is communicated as an additional state space dimension to the agent. The agent uses this task information and the reward *r*_*e*_ for learning the task policy π_*e*_ but ignores those information when improving π_*o*_ such that skills remain reusable in different tasks. The exploration option *o*_*e*_ used in the developmental period was removed from the skill set *O* such that the agent can only choose among self-discovered skills.

**Figure 7:**
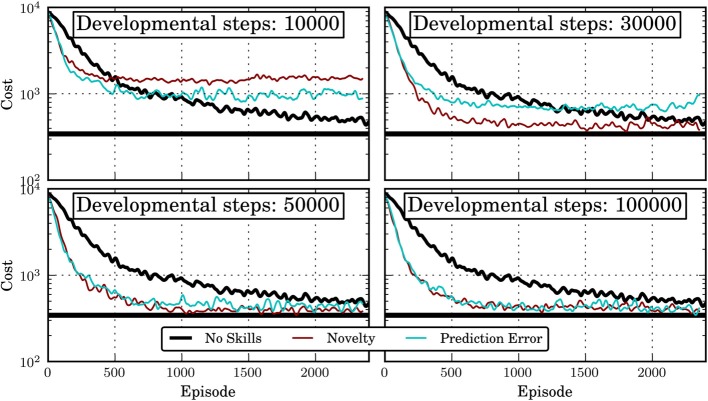
**Cost (negative return) of IMRL agent in the 12 tasks 2D Multi-Valley domain for different intrinsic motivation systems and different lengths of the developmental period**. “No Skills” shows the performance of a monolithic agent that does not learn skills and has no developmental period. The horizontal black line shows the average cost of the policy learned by the monolithic agent after 5000 episodes. Shown is the mean over 10 independent runs that have been smoothened by a moving window average with window length 50.

Figure 7 shows the results for different intrinsic motivation mechanisms and different lengths of the developmental period. As baseline, “No Skills” shows the performance of an agent that learns a monolithic policy for each task separately. For a very short developmental period of 10,000 steps, the hierarchical agent, which uses skills learned in the developmental period, learns initially faster than the monolithic agent, however, it converges to considerably worse policies. This is probably due to the fact that not all relevant skills have been discovered in the developmental period. See Jong et al. (2008) for a discussion of why an incomplete set of skills might have a detrimental effect on an agent's performance. For 30,000 developmental steps, the skills acquired under the novelty motivation allow already to achieve close-to-optimal performance while the ones from the prediction-error motivation do not. This corresponds to the different qualities of the learned skills under the two motivation systems (compare Figure 5). For 50,000 or more developmental steps, the performance of the hierarchical agent approaches the optimal performance considerably faster than the monolithic agent, irrespective of the intrinsic motivation system used. This is interesting since after 50,000 steps, the learned skills are far from optimal (compare Figure 5). Apparently, also skills with sub-optimal policies can help the agent considerably. It should also be noted that even though a close-to-optimal performance is reached relatively fast, the performance remains slightly below the optimum which is reached by the monolithic agent after 5000 episodes. This is probably due to the (temporal) abstraction introduced by the skills which on the one hand helps the agent in learning faster but on the other hand also reduces the class of representable policies.

### 4.2. Octopus

#### 4.2.1. Problem domain

**Figure 8:**
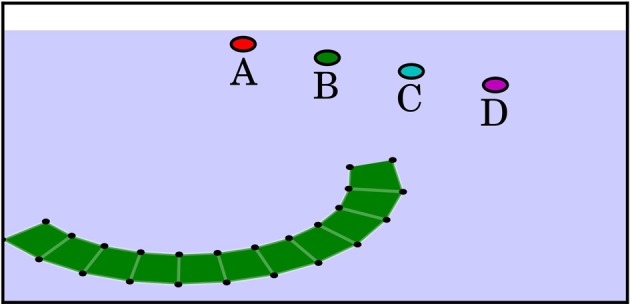
**Visualization of the octopus arm task**. The circles represent target objects used in different tasks which yield an external reward when touched.

In the octopus arm domain^3^ (Yekutieli et al., 2005), the agent has to learn to control an Octopus arm. The base of the arm is restricted and cannot be actuated directly. The agent may control the arm in the following way: elongating or contracting the entire arm, bending the first half of the arm in either of the two directions, and bending the second half of the arm in either of the two directions. In each time step, the agent can set the elongation and the bending of the first and second half of the arm to an arbitrary value in [−1, 1], resulting in 3 continuous action dimensions. The agent observes the positions *x*_*i*_, *y*_*i*_ and velocities ẋ_*i*_, ẏ_*i*_ of 24 selected parts of its arm (denoted by small black dots in Figure 8) and the angle and angular velocity of the arm's base. Thus, the state space is continuous and consists of 98 dimensions. Because of the high-dimensional and continuous state and action spaces and the complex dynamics of the domain, the octopus arm problem is a challenging task. It can also be seen as an easy simulation-based benchmark for actual robotic manipulation tasks.

#### 4.2.2. Developmental period

Similar to the developmental period in the 2D multi-valley domain, the agent can explore the domain freely while engaging in skill discovery and following its intrinsic motivations. However, the basis for skill discovery is not to identify bottlenecks (there are no bottlenecks in this domain) but to cluster the transition graph into regions which correspond to similar qualitative states. Thus, a different linkage criterion *l*_*G*_ has been used: for two subgraphs *A* and *B* of the transition graph *G*, the linkage is set to *l*_*G*_(*A, B*) = 1/| *A* ∪ *B*|^2^∑_*v, v*′ ∈ *A* ∪ *B*_ *d*_*sp*_(*v, v*′), i.e., the average length of the shortest paths *d*_*sp*_ between two nodes in *A* ∪ *B*. This linkage results in clusters with similar states in the sense that the agent can traverse from one state of the cluster to the other with a small number of steps. The maximum linkage ψ of a cluster in OGAHC has been set to 3.0 and skill discovery with OGAHC was performed every 10,000 steps. The greediness of IFIGE has been set to α_*i*_ = 0.25 and the split node distance to ζ = 7.5. Intrinsic motivation was based on the novelty mechanism with *b* = 1 and the length of the developmental period was set to 50,000 steps.

Because of the continuous action space, we have used direct policy search based on evolutionary computation for learning option policies π_*o*_. The value for *j*-the action dimension is determined via *a*_*j*_ = tanh(∑^98^_*k* = 0_ *w*_*jk*_*s*_*k*_), where *s*_*k*_ is the value of the *k*-th state dimension and *s*_98_ = 1 is a bias. The policy's weights *w*_*jk*_ have been optimized using 16 + 40 evolution strategy (ES) and each weight vector has been evaluated 10 times. The pseudo-reward for each option's policy has been set to *r*_*o*_ = −1 for each step and *r*_*o*_ = −100 if an option terminates unsuccessfully. The ES' objective is to maximize the pseudo-reward accumulated in 10 steps, after which the option is interrupted.

As in the multi-valley domain, the agent has initially only a single option *o*_*e*_ in its skill pool *O*, which can be invoked in any state of the environment, i.e., *I*_*o*_*e*__ = *S*, and terminates with probability β_*o*_*e*__(*s*) = 0.1. π_*o*_*e*__ selects actions uniform randomly from the action space. The higher-level policy π_*i*_, which determines the option that is executed, has been learned using Q-Learning with discounting factor γ = 0.99 and exploration rate ϵ = 0.01. Because of the high dimensionality of the state space, the value function was not represented using a CMAC function approximator but using a linear combination of state values, i.e., *Q*(*s, o*) = ∑^98^_*k* = 0_*w*_*o*__*k*_*s*_*k*_. The learning rate has been set to 0.1.

#### 4.2.3. Task performance

Different tasks can be imposed onto the agent; in this work, we require that the agent learns to reach for certain objects that are located at different positions (compare Figure 8). The agent obtains an external reward of −0.01 per time step and a reward of 100 for reaching the target object. The episode ends after 1000 time steps or once the target object is reached.

**Figure 9:**
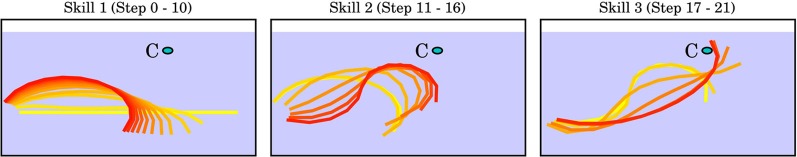
**Example trajectory of the octopus arm controlled by the IMRL agent**. The trajectory corresponds to a sequence of three skills. Yellowly colored arms correspond to states at the beginning of skill execution while redly colored arms correspond to states at the end of skill execution.

Figure 9 depicts an example trajectory of the octopus arm learned by the IMRL agent for reaching a target located at position C: the goal is reached after 22 steps and the agent invokes three different skills during this trajectory. The skill executed in the first 11 steps contracts the arm and brings it into an ∩-shape. The skill chosen for the next 6 steps unrolls the first part of the arm until an S-shape is reached. The skill executed in the last 5 steps unrolls the second half of the arm such that the target object is reached by an ∪-shape. Note that bending the arm directly into an ∪-shape would not be successful but result in a state like the one depicted in Figure 8.

**Figure 10:**
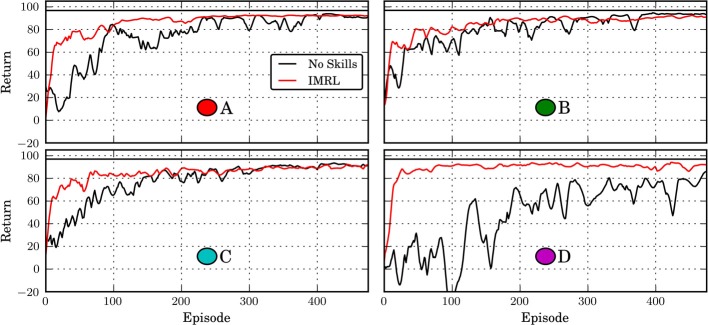
**Return of IMRL agent in the Octopus domain under the “novelty” motivation after 50,000 developmental steps**. The circle patches indicate the respective targets used in the runs (compare Figure 8). “No Skills” shows the performance of an agent that does not learn skills and has no developmental period. The horizontal black line shows the average cost of the policy learned by the monolithic agent after 2500 episodes. All curves show median performance over 5 independent runs and have been smoothened by a moving window average with window length 25.

Figure 10 shows the learning curves of the IMRL agent and a monolithic agent, which learns a flat global policy with the same parametrization as the skill policies, for different target positions in the Octopus domain. Given sufficient time, the monolithic agent can learn policies of similar quality as the IMRL agent. Thus, close-to-optimal behavior can be represented by a flat global policy. However, in general, the IMRL agent learns close-to-optimal policies faster and the learning curves exhibit less variance across all tasks. Thus, the temporal abstraction of the skills that were learned in the developmental period seem to make learning close-to-optimal behavior easier by providing a useful explorative bias. On the other hand, as in the multi-valley domain these abstractions may impair performance slightly in the long run.

## 5. Conclusion and future work

We have presented a novel skill discovery approach suited for continuous domains that can be used by an IMRL agent in its developmental period. Our empirical results in two continuous RL domains suggest that the IMRL agent benefits from the discovered skills once it is faced with external tasks: close-to-optimal behaviors can be learned in less trials because of the explorative bias provided by the temporal abstractions of the skill hierarchy. However, this explorative bias is only helpful if the developmental period was sufficiently long: if the learning and discovery of skills is interrupted prematurely, an IMRL agent might perform worse than an agent which learns a monolithic policy from scratch. Furthermore, we have compared two intrinsic motivation mechanisms and presented evidence that intrinsic motivation allows to reasonably determine how much time should be spend on learning specific skills.

This work can be extended in numerous ways: for instance, instead of performing skill discovery only in the developmental period, the agent could also discover novel skills and learn based on intrinsic motivation while he is faced with an external task. This, however, requires trading off intrinsic and external rewards and facing the exploration-exploitation dilemma. We leave this to future work; however, we would like to emphasize that the proposed skill discovery approach is in no way restricted to the developmental setting. A further direction of future work would be to combine the proposed skill discovery approach with more sophisticated intrinsic motivation mechanisms such as competence progress intrinsic motivation (Stout and Barto, 2010) or other means for empirically estimating the learning progress (see, e.g., Lopes et al., 2012). Furthermore, it would be desirable to learn more complex hierarchies of skills, where skills can invoke other skills. The dendrogram generated by the hierarchical clustering in OGAHC could be an interesting starting point for this. For being useful in a realistic robotic setup, the proposed methods would need to be integrated into a control architecture with, e.g., reactive behaviors and predictive control, such as the one shown in Figure 1. This should allow to deal better with non-markovian, noisy, and partial observable problems.

### Conflict of interest statement

The authors declare that the research was conducted in the absence of any commercial or financial relationships that could be construed as a potential conflict of interest.

## Footnotes

^1^In order to keep the size of *S*_*o*_ limited, we remove states from *S*_*o*_ once |*S*_*o*_| > 2500. The heuristic for selecting the state that is removed is to remove one of the states of the (approximate) closest state pair in *S*_*o*_. This results in covering the effective state space of the problem approximately uniform.

^2^The floor of valley 0 (see Figure 3) corresponds to the region ((−1/6 ± 2/15)π, (−1/6 ± 2/15)π).

^3^Source code available via http://cs.mcgill.ca/dprecup/workshops/ICML06/octopus.html.

## References

[B1] Baldassarre G. (2011). What are intrinsic motivations? A biological perspective, in IEEE International Conference on Development and Learning. Vol. 2 (Frankfurt am Main), 1–8 10.1109/DEVLRN.2011.6037367

[B2] Barto A. G. Mahadevan S. (2003). Recent advances in hierarchical reinforcement learning. Dis. Event Dyn. Syst. 13, 341–379 10.1023/A:1022140919877

[B3] Barto A. G. Singh S. Chentanez N. (2004). Intrinsically motivated learning of hierarchical collections of skills, in Proceedings of the 3rd International Conference of Developmental Learning (LaJolla, CA), 112–119

[B4] Harlow H. F. (1950). Learning and satiation of response in intrinsically motivated complex puzzle performance by monkeys. J. Compar. Physiol. Psychol. 43, 289–294 10.1037/h0058114 15436888

[B5] Hester T. Stone P. (2012). Intrinsically motivated model learning for a developing curious agent, in Proceedings of the 11th International Conference on Development and Learning (San Diego, CA). 10.1109/DevLrn.2012.6400802

[B6] Jong N. K. Hester T. Stone P. (2008). The utility of temporal abstraction in reinforcement learning, in Proceedings of the 7th Conference on Autonomous Agents and Multiagent Systems (Estoril), 299–306

[B7] Kirchner F. (1998). Q-learning of complex behaviours on a six-legged walking machine. J. Robot. Auton. Syst. 25, 256–263 10.1016/S0921-8890(98)00054-2

[B8] Kirchner F. Richter C. (2000). Q-surfing: exploring a world model by significance values in reinforcement learning tasks, in Proceedings of the European Conference on Artificial Intelligence (Berlin), 311–315

[B9] Köhler T. Rauch C. Schröer M. Berghöfer E. Kirchner F. (2012). Concept of a biologically inspired robust behaviour control system, in Proceedings of 5th International Conference on Intelligent Robotics and Applications (Montreal, QC), 486–495 10.1007/978-3-642-33515-0_48

[B10] Konidaris G. Barto A. G. (2009). Skill discovery in continuous reinforcement learning domains using skill chaining, in Advances in Neural Information Processing Systems (NIPS). Vol. 22 (Vancouver, BC), 1015–1023

[B11] Lopes M. Lang T. Toussaint M. Oudeyer P.-Y. (2012). Exploration in model-based reinforcement learning by empirically estimating learning progress, in Advances in Neural Information Processing Systems (NIPS) (Lake Tahoe, Nevada), 206–214

[B12] Mannor S. Menache I. Hoze A. Klein U. (2004). Dynamic abstraction in reinforcement learning via clustering, in Proceedings of the 21st International Conference on Machine Learning (Banff, AB), 560–567 10.1145/1015330.1015355

[B13] McGovern A. Barto A. G. (2001). Automatic discovery of subgoals in reinforcement learning using diverse density, in Proceedings of the 18th International Conference on Machine Learning (Williamstown, MA), 361–368

[B14] Menache I. Mannor S. Shimkin N. (2002). Q-Cut – dynamic discovery of sub-goals in reinforcement learning, in Proceedings of the 13th European Conference on Machine Learning (Helsinki, Finland), 295–306 10.1007/3-540-36755-1_25

[B15] Metzen J. H. (2012). Online skill discovery using graph-based clustering. J. Mach. Learn. Res. W&CP 24, 77–88

[B15a] Metzen J. H. (in press). Learning graph-based representations for continuous reinforcement learning domains, in Proceedings of the European Conference on Machine Learning, (ECML 2013), (Prague: Springer).

[B16] Oudeyer P.-Y. Kaplan F. (2007). What is intrinsic motivation? A typology of computational approaches. Front. Neurorobot. 1:6 10.3389/neuro.12.006.2007 18958277 PMC2533589

[B17] Oudeyer P.-Y. Kaplan F. Hafner V. (2007). Intrinsic motivation systems for autonomous mental development. IEEE Trans. Evol. Comput. 11, 265–286 10.1109/TEVC.2006.890271

[B18] Randløv J. Alstrøm P. (1998). Learning to drive a bicycle using reinforcement learning and shaping, in Proceedings of the 15th International Conference on Machine Learning (Madison, WI), 463–471

[B19] Schembri M. Mirolli M. Baldassarre G. (2007). Evolution and learning in an intrinsically motivated reinforcement learning robot, in Proceedings of the 9th European Conference on Advances in Artificial Life (Lisbon, Portugal), 294–303 10.1007/978-3-540-74913-4_30

[B20] Schmidhuber J. (1991). Curious model-building control systems, in Proceedings of the International Joint Conference on Neural Networks (Singapore: IEEE), 1458–1463

[B21] Şimşek Ö. Barto A. G. (2004). Using relative novelty to identify useful temporal abstractions in reinforcement learning, in Proceedings of the 21st International Conference on Machine Learning (Banff, AB), 751–758 10.1145/1015330.1015353

[B22] Şimşek Ö. Barto A. G. (2009). Skill characterization based on betweenness, in Advances in Neural Information Processing Systems (NIPS). Vol. 22 (Vancouver, BC), 1497–1504

[B23] Şimşek Ö. Wolfe A. P. Barto A. G. (2005). Identifying useful subgoals in reinforcement learning by local graph partitioning, in Proceedings of the 22nd International Conference on Machine Learning (Bonn, Germany), 816–823 10.1145/1102351.1102454

[B24] Skinner B. (1938). The Behavior of Organisms: An Experimental Analysis. The Century Psychology Series. New York, NY: Appleton-Century-Crofts

[B25] Stout A. Barto A. G. (2010). Competence progress intrinsic motivation, in Proceedings of the 9th IEEE International Conference on Development and Learning (Ann Arbor, MI), 257–262 10.1109/DEVLRN.2010.5578835

[B26] Sutton R. S. Barto A. G. (1998). Reinforcement Learning: An Introduction. Cambridge, MA: The MIT Press

[B27] Sutton R. S. Koop A. Silver D. (2007). On the role of tracking in stationary environments, in Proceedings of the 24th International Conference on Machine Learning (Corvallis, OR: ACM), 871–878 10.1145/1273496.1273606

[B28] Sutton R. S. Precup D. Singh S. (1999). Between MDPs and semi-MDPs: a framework for temporal abstraction in reinforcement learning. Artif. Intell. 112, 181–211 10.1016/S0004-3702(99)00052-1

[B29] Tenenbaum J. B. Silva V. D. Langford J. C. (2000). A global geometric framework for nonlinear dimensionality reduction. Science 290, 2319–2323 10.1126/science.290.5500.2319 11125149

[B30] Thrun S. (1996). Is learning the n-th thing any easier than learning the first? in Advances in Neural Information Processing Systems (NIPS) (Cambridge, MA: MIT Press), 640–646

[B31] Whiteson S. (2012). Evolutionary computation for reinforcement learning, in Reinforcement Learning: State of the Art (Berlin: Springer), 325–358

[B32] Yekutieli Y. Sagiv-Zohar R. Aharonov R. Engel Y. Hochner B. Flash T. (2005). A dynamic model of the octopus arm. I. Biomechanics of the octopus reaching movement. J. Neurophysiol. 5, 291–323 15829594 10.1152/jn.00684.2004

